# Supplementary material for: Loss of CFTR function is associated with reduced bitter taste receptor-stimulated nitric oxide innate immune responses in nasal epithelial cells and macrophages
Source: Front Immunol. 2023 Jan 18;14:1096242. doi: 10.3389/fimmu.2023.1096242 (PMC9890060; doi:10.3389/fimmu.2023.1096242)
Supplement: Supplementary file 1 [file DataSheet_1.pdf]

Supplemental Figure 1

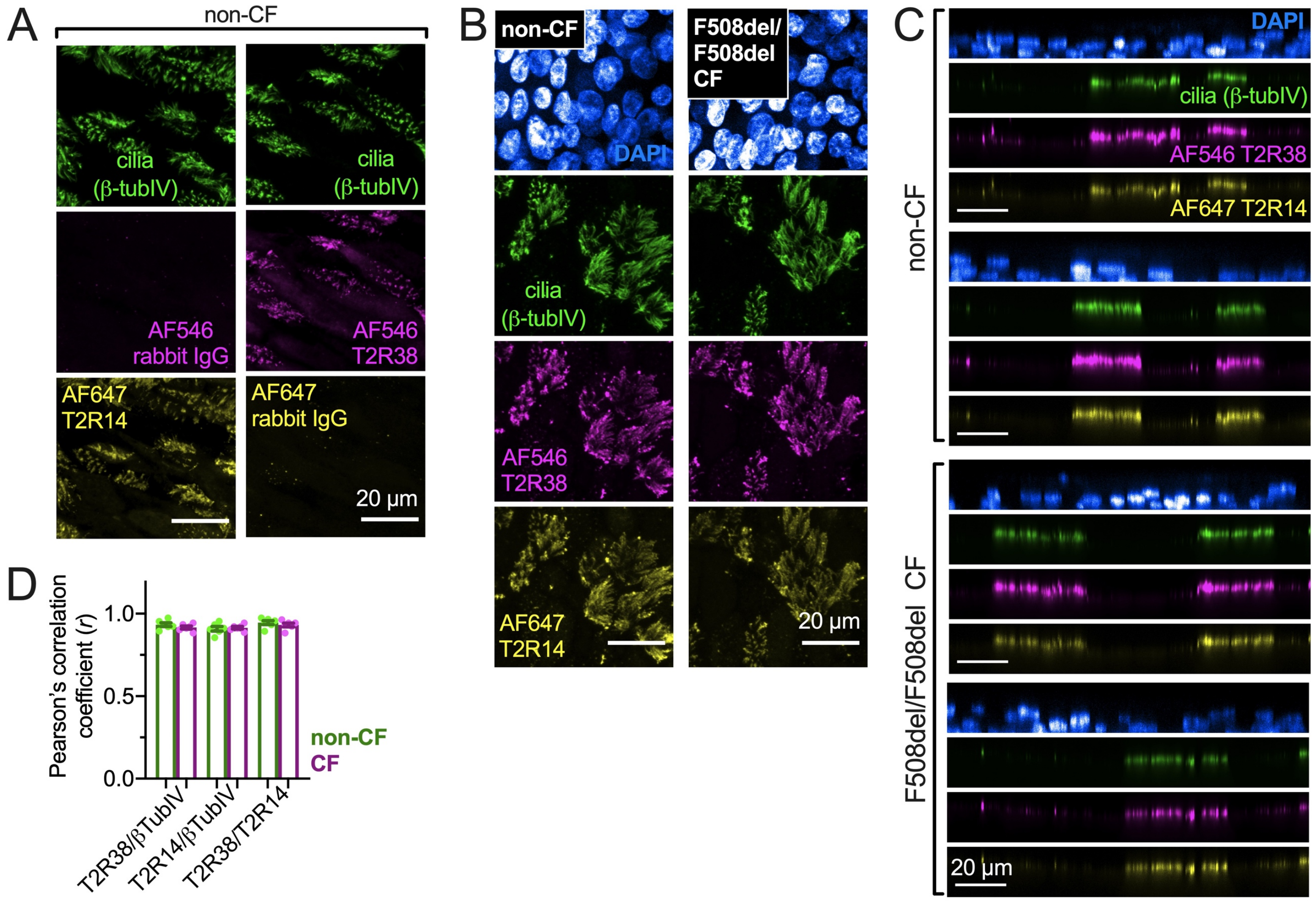

Supplemental Figure 1. Immunofluorescence localization of T2R38 and T2R14 in CF and non-CF nasal cilia. (A) Representative images showing staining of two non-CF patient ALIs with AlexaFluor (AF) 546 labeled T2R38 primary antibody, AF647 labeled T2R14 primary antibody, or AF546 or AF647 labeled rabbit serum control. Representative of results from 3 independent experiments using cells from 3 non-CF patients. **(B)** Representative images comparing staining of AF546-labeled T2R38 antibody and AF647-labeled T2R14 antibody in non-CF (left) and CF (right) patient ALIs. **(C)** Orthogonal plans from images as in *C*, showing similar localization in CF and non-CF cells. Images taken at 60x 1.4NA objective with 0.2 μm *z* step size, not corrected for refractive index mismatch to minimize image processing. Images are representative of 3 independent experiments using cells from 3 CF and 3 non-CF patients. **(D)** Pearson's correlation coefficient was computed using ImageJ showing strong correlation ($r \geq 0.9$) of the T2R14 and T2R38 signals as well as either T2R14 and T2R38 signals with β-tubulin IV. No significant differences were observed between CF and non-CF patients by ANOVA. All results shown are representative of 4 ALIs from 4 individual CF and 4 individual non-CF patients.

Supplemental Figure 2

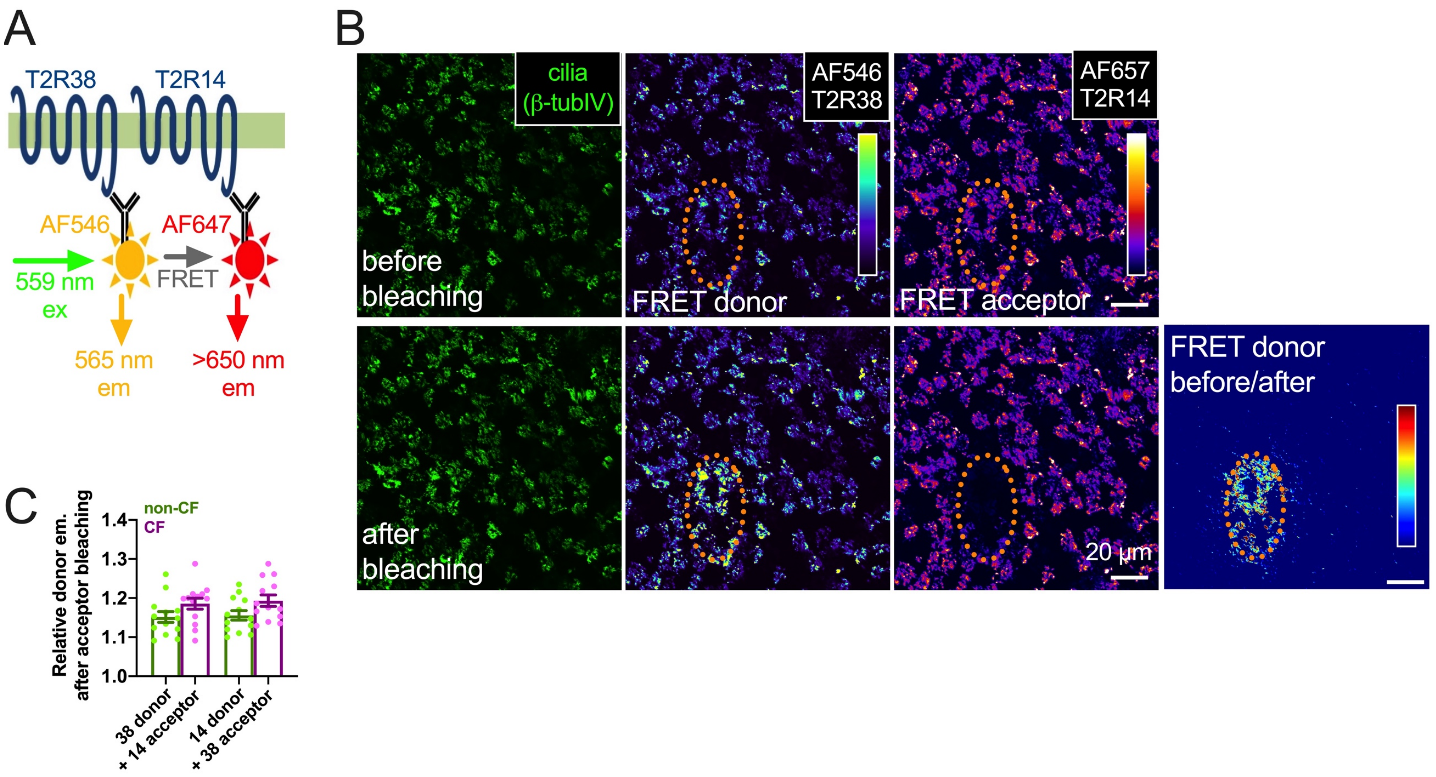

Supplemental Figure 2. Confirmation of similar co-localization of T2R14 and T2R38 by quantification of antibody-based Förster resonance energy transfer (FRET). (A) Co-localization was quantified by measurement of FRET efficiency of labeled primary antibodies as described in (1, 2). **(B)** Bleaching of a region of the FRET acceptor via 647 laser and tornado bleaching function in Olympus Fluoview (photo-bleached area shown in yellow circle) resulted in an increase in fluorescence of the donor, confirming FRET. **(C)** Quantification of FRET efficiency (the relative increase in donor fluorescence after acceptor bleaching; E). as described in the methods revealed similar FRET between T2R38 and T2R14 CF and non-CF cells. Results representative of 12 independent experiments using ALIs grown from 4 CF and 4 non-CF patients (3 ALIs each). E was not significantly different between the two genotypes. As described (1), FRET efficiency decreases to the 6th power as distance (R) increases, thus $R = R_o \bullet [(1\text{-}E)/E]^{1/6}$ where R_o is the distance at which the E is 50% (51 Å for AF555 and AF647). A ~0.16 E equates to ~67 Å in distance between the two fluorophores. Together with Figure 2 in the main text, these data suggest no major changes in T2R localization between CF and non-CF airway epithelial cells.

Supplemental Figure 3

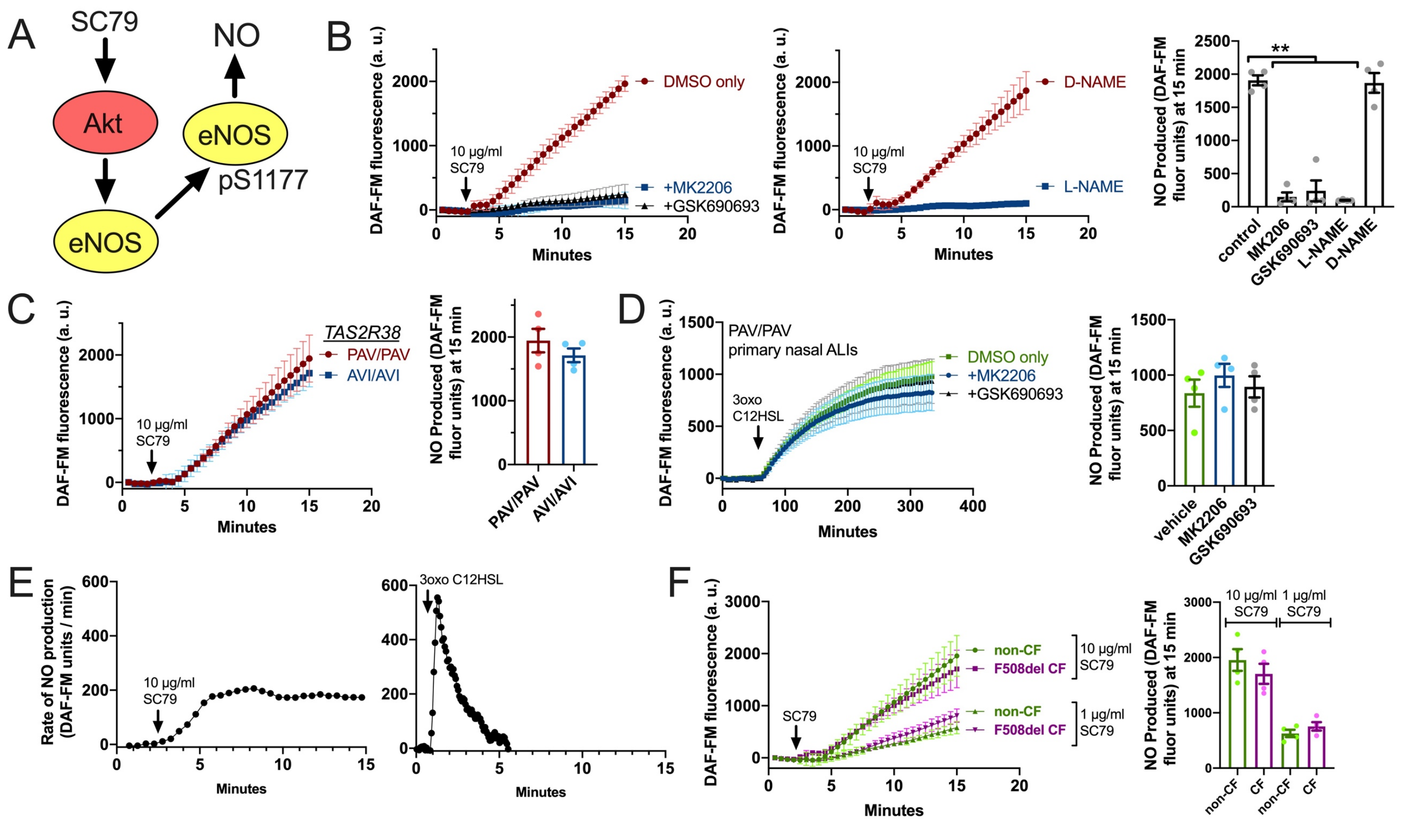

Supplemental Figure 3. Intact Akt-stimulated NO production in CF nasal epithelial cells. (A) It was previously demonstrated (3) that Akt activator SC-79 causes eNOS phosphorylation at S1177 in airway epithelial cells, stimulating NO production. **(B)** Left and middle, traces of DAF-FM fluorescence increases in response to 10 μg/ml SC79 showing increased in NO production that is inhibited by Akt inhibitors MK2206 and GSK690693 (left; 1 μM each for 45 min pre-treatment) or NOS inhibitor L-NAME (right; 10 μM for 45 min pre-treatment) but not inactive analogue D-NAME. Right is bar graph showing data from independent experiments using cells from different non-CF individuals. Significance by one-way ANOVA with Dunnett's posttest comparing all values to control; ***p*<0.01 **(C)** SC79/Akt-stimulated NO production is not different in cells genotyped for *TAS2R38* PAV/PAV or AVI/AVI, confirming that the SC79 response is independent of T2R38 function. No significant difference by Student's *t* test. **(D)** Trace (left) and bar graph of independent experiments using ALIs from different patients (right) of DAF-FM fluorescence in PAV/PAV *TAS2R38* genotyped cells with 3oxoC12HSL (T2R38 agonist; 100 μM). No inhibition was observed with Akt inhibitors MK2206 or GSK690693, confirming T2R38 activation of eNOS is independent of Akt. No significant difference by one way ANOVA. **(E)** Forward derivative of representative DAF-FM fluorescence (showing rate of DAF-FM fluorescence change, roughly equating relative rates of NO production) during stimulation with SC79 or 3oxoC12HSL (100 μM) showing different kinetics of rate of NO production. SC79 activated more sustained NO production while T2R stimulation activated more of a burst of NO production that tapered off. **(F)** Representative traces (left) and bar graph of independent experiments using cells from different patients (right) showing no difference between CF and non-CF cells in terms of NO production with two different concentrations of SC79. No significant differences by one-way ANOVA. Together, these data suggest that Akt-activated NO production is intact in CF cells, and suggest there is a specific defect in T2R-activated NO production rather than a general defect in the ability of CF cells to produce NO. These data also support that the DAF-FM dye itself is not exhibiting different sensitivity in CF vs non-CF cells underlying the changes observed in the main text.

Supplemental Figure 4

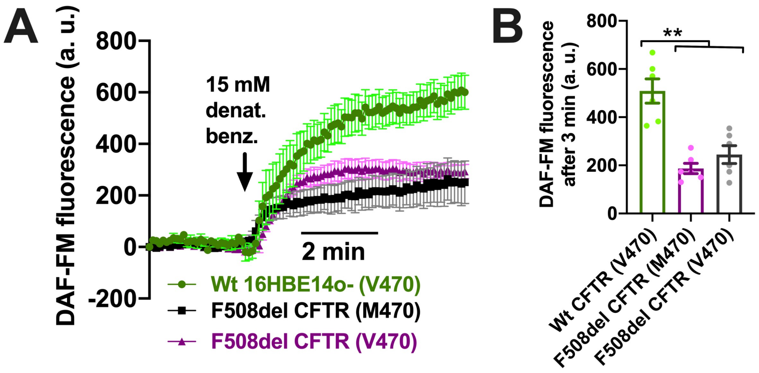

Supplemental Figure 4. DAF-FM fluorescence changes were reduced in both F508del M470 and V470 cells. (A) DAF-FM fluorescence traces showing increases in responses to multi-T2R agonist denatonium benzoate in cells with Wt CFTR, F508del M470, or F508del V470, generated as described (4). **(B)** Bar graph showing data from independent experiments (n = 5) showing reduced DAF-FM fluorescence change in both F508del M470 and V470, which were not significantly different from each other. Significance by one way ANOVA with Bonferroni posttest; ***p*<0.01.

Supplemental material for Carey, *et al.*, "Bitter taste receptor-stimulated nitric oxide innate immune responses are reduced by loss of CFTR function in nasal epithelial cells and macrophages."

Supplemental Figure 5

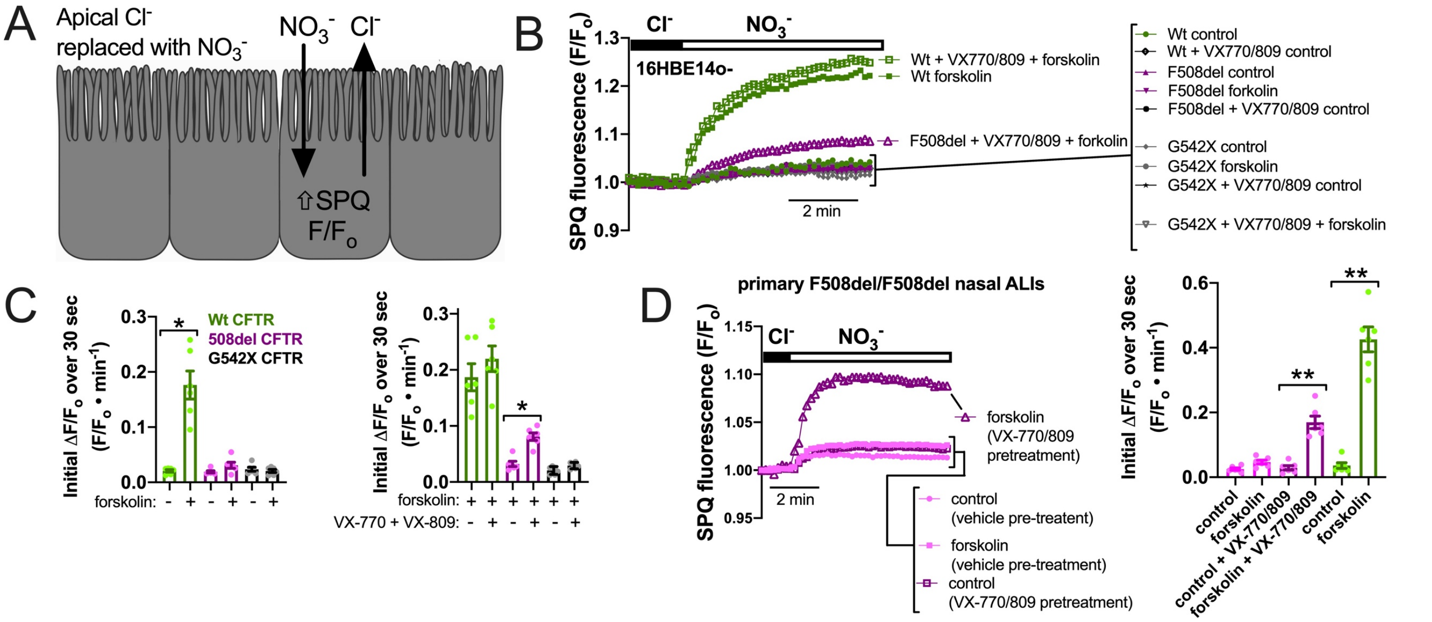

Supplemental Figure 5: Increased apical Cl⁻ permeability in CF cells treated with VX-770+VX-809. (A) Diagram of NO substitution assay (used in (5)). SPQ is quenched by Cl⁻ but not by NO_3^-. Most Cl⁻ channels, including CFTR, have a nearly equal permeability to Cl⁻ and NO_3^-. Apical substitution of NO_3^- for Cl⁻ results in electroneutral efflux of Cl⁻ and influx of NO_3^- along diffusional gradients, resulting in an increase in intracellular SPQ fluorescence (expressed as fluorescence normalized to fluorescence at time 0 [F/F_o]). **(B)** Representative traces of SPQ during apical NO_3^- substitution ± forskolin stimulation, which resulted in an increase in the rate of SPQ fluorescence change (roughly equal to an increase in the relative apical anion permeability) in 16HBE14o- cells with Wt (parental strain), F508del (CRISPR modified), G542X (CRISPR modified) CFTR (4). **(C)** Bar graph from experiments as in *B* showing data points of initial rate of SPQ fluorescence change from 5-7 independent experiments. Significance by one-way ANOVA with Bonferroni posttest; *p<0.05. Non-CF (Wt CFTR parental 16HBEs) cells showed an increase in the rate of SPQ fluorescence change with forskolin stimulation. F508del and G542X CFTR cells did not. VX-770+VX-809 pre-treatment resulted in a significant increase in forskolin-stimulated SPQ change in F508del but not G542X cells. **(D)** Trace and bar graph showing experiments similar to *B* and *C* except using primary nasal ALIs from F508del/F508del CF patients. CF patients shown in pink. Bar graph also shows non-CF experiments shown in green, which exhibited an increase in SPQ fluorescence change with forskolin stimulation. F508del/F508del cells only showed a forskolin-stimulated increase after pre-treatment with VX-770+VX-809. These results confirm that VX-770+VX-809 increased apical anion permeability in F508del but not G542X cells, suggesting corrector/potentiator treatment increased F508del CFTR activity.

Supplemental Figure 6

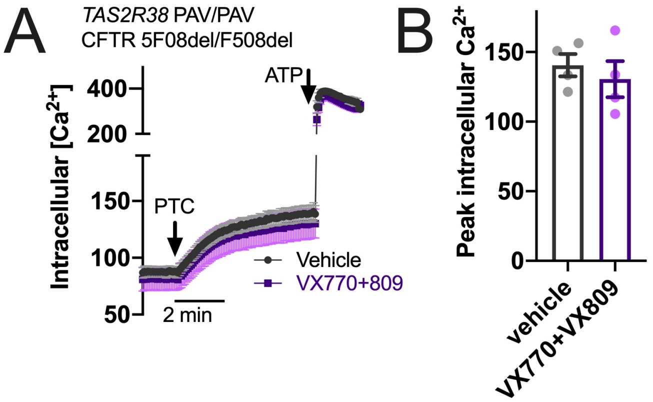

Supplemental Figure 6. PTC-evoked Ca^{2+} increases were not different in *TAS2R38* PAV/PAV CF ALIs after pre-treatment with V-X770+VX-809. (A) Cells were loaded with Fura-2 identically as described in the text for Fluo-4 and as in (1). Trace showing intracellular Ca^{2+} (calibrated from Fura-2 340/380 ratio as described in (1) using the method of Grynkiewicz (6)). Both resting Ca^{2+} and stimulated Ca^{2+} appeared the same. **(B)** Bar graph showing peak PTC-stimulated Ca^{2+} from independent 4 experiments using ALIs from 4 different CF PAV/PAV patients. No significant difference by one-way ANOVA. These data suggest that VX-770+VX-809 pretreatment is not increasing T2R-stimulated NO production in CF cells due to enhancement of T2R Ca^{2+} signaling.

Supplemental material for Carey, *et al.*, “Bitter taste receptor-stimulated nitric oxide innate immune responses are reduced by loss of CFTR function in nasal epithelial cells and macrophages.”

Supplemental Table 1

Patient	Age at Surgery	Gender	Ethnicity	Diagnosis	# Prior FESS	Polyps	Lund-Mackay	SNOT-22	Smoking History	Asthma	AFS	Abx	Steroids	Comorbidities	CFTR genotype
CF1	25	Female	Caucasian	CF, CRS	1	No	18	67	No	Yes	No	Yes	Yes	CF, GERD, Asthma	F508del/1833delT
CF2	28	Female	Caucasian	CF, CRS	1	No	10	78	No	Yes	No	Yes	Yes	CF, Asthma, GERD, DM	F508del/F508del
CF3	38	Female	Caucasian	CF,CRS	1	Yes	16	93	No	No	No	No	Yes	CF, Lung transplant, DM	F508del/F508del
CF4	42	Male	Caucasian	CF, CRS	1	No	14	33	No	Yes	No	Yes	No	CF, Lung transplant, DM, GERD, HTN	F508del/E585X
CF5	26	Female	Caucasian	CF, CRS	1	No	N/A	78	No	Yes	No	No	No	CF, GERD, DM	F508del/F508del
CF6	23	Male	Caucasian	CF, CRS	1	Yes	N/A	N/A	No	No	No	No	No	CF, GERD	F508del/F508del
CF7	33	Female	Caucasian	CF, CRS	1	Yes	13	8	No	No	No	No	No	CF, Lung Transplant, Allergies, GERD, HTN, DM	F508del/F508del
CF8	32	Female	Caucasian	CF, CRS	1	Yes	N/A	77	No	Yes	No	No	No	CF, GERD	F508del/F508del
CF9	58	Female	Caucasian	CF, CRS	2	Yes	18	27	No	Yes	No	No	No	Allergies, GERD, HTN	F508del/F508del
CF10	27	Female	Caucasian	CF, CRS	0	No	N/A	59	No	Yes	No	No	No	CF	F508del/G542X
CF11	32	Male	Caucasian	CF, CRS	2	Yes	N/A	43	No	Yes	No	No	Yes	CF	F508del/F508del
CF12	38	Male	Caucasian	CF, CRS	1	No	N/A	28	No	No	No	No	No	CF, Allergies	F508del/F508del
CF13	22	Male	Caucasian	CF, CRS	9	Yes	20	15	No	No	No	No	Yes	CF, Allergies, GERD, DM	F508del/F508del
CF14	41	Male	Caucasian	CF, CRS	0	Yes	14	32	No	Yes	No	Yes	No	CF, Allergies, DM	F508del/F508del

Patient	Age at Surgery	Gender	Ethnicity	Diagnosis	# Prior FESS	Polyps	Lund-Mackay[1,2]	SNOT-22[3]	Smoking History	Asthma	AFS	Abx	Steroids	Comorbidities	CFTR genotype
nonCF1	37	Male	Caucasian	CRS	1	Yes	18	68	Yes	No	No	No	No	Allergies, Sinonasal Trauma	N/A
nonCF2	24	Female	Caucasian	CRS	0	No	13	69	No	No	No	No	No	Allergies, GERD	N/A
nonCF3	19	Male	Caucasian	CRS	1	Yes	N/A	41	No	Yes	No	No	No	Allergies	N/A
nonCF4	21	Male	Caucasian	Orbital decompression	0	No	0	0	Yes	No	No	No	No	N/A	N/A
nonCF5	32	Male	Caucasian	CRS	1	Yes	10	42	No	No	No	No	Yes	Allergies, GERD	N/A
nonCF6	34	Female	Caucasian	CRS	0	Yes	6	34	No	Yes	No	No	No	Allergies	N/A
nonCF7	28	Female	Caucasian	CRS	1	Yes	26	27	No	No	No	No	Yes	DM	N/A
nonCF8	43	Male	Caucasian	CRS	2	Yes	20	58	No	No	Yes	Yes	Yes	AERD, Allergies, HTN	N/A
nonCF9	39	Male	Caucasian	CRS	1	Yes	16	59	No	Yes	No	No	Yes	DM	N/A
nonCF10	44	Male	Caucasian	CRS	2	Yes	7	22	No	No	No	Yes	No	N/A	N/A
nonCF11	44	Female	Caucasian	CRS	0	Yes	20	30	No	Yes	No	No	No	HTN, Diabetes	N/A
nonCF12	44	Male	Caucasian	CRS	1	No	12	24	No	No	No	No	No	HTN	N/A
nonCF13	28	Male	Caucasian	Sinonasal tumor	0	No	4	40	Yes	No	No	No	No	N/A	N/A
nonCF14	32	Male	Caucasian	CRS	0	No	3	53	No	No	No	No	No	N/A	N/A
nonCF15	28	Male	Caucasian	CRS	1	No	3	N/A	No	No	No	No	No	Allergies	N/A
nonCF16	21	Female	Caucasian	CRS	0	No	3	14	No	No	No	No	No	GERD	N/A
nonCF17	44	Female	Caucasian	CRS	0	No	4	40	Yes	No	No	No	No	Allergies	N/A
nonCF18	42	Female	African American	CRS	1	No	1	89	No	Yes	No	No	No	Asthma, Allergies, PE	N/A
nonCF19	35	Female	Caucasian	CRS	0	No	4	N/A	No	No	No	No	No	N/A	N/A
nonCF20	23	Female	Caucasian	CRS	0	Yes	N/A	39	No	Yes	No	No	No	N/A	N/A
nonCF21	37	Female	Caucasian	CRS	2	Yes	18	80	No	Yes	No	No	Yes	GERD, Allergies	N/A
nonCF22	52	Female	Caucasian	CRS	1	No	5	20	No	No	No	No	No	N/A	N/A
nonCF23	58	Male	Caucasian	CRS	0	No	11	25	No	No	Yes	No	No	GERD, Allergies	N/A

Supplemental Table 1. Clinical characteristics of CF (top) and non-CF (bottom) patients from whom nasal tissue was used, including Lund-Mackay (7, 8) and SNOT-22 (9) scores, both reflecting the severity of sinonasal disease symptoms. Abbreviations: Abx, history of antibiotics; AFS, allergic fungal sinusitis; ARS, allergic rhinosinusitis; CRS, chronic rhinosinusitis; DM, diabetes mellitus; FESS, functional endoscopic sinus surgery; GERD, gastroesophageal reflux disease; HTN, hypertension; N/A, not available; SNOT-22, 22 question sinonasal outcomes test.

Supplemental References

1. Hariri BM, McMahon DB, Chen B, Freund JR, Mansfield CJ, Doghramji LJ, et al. Flavones Modulate Respiratory Epithelial Innate Immunity: Anti-Inflammatory Effects and Activation of the T2r14 Receptor. *J Biol Chem* (2017) 292(20):8484-97. Epub Apr 3. doi: 10.1074/jbc.M116.771949.

2. Konig P, Krasteva G, Tag C, Konig IR, Arens C, Kummer W. Fret-Clsm and Double-Labeling Indirect Immunofluorescence to Detect Close Association of Proteins in Tissue Sections. *Lab Invest* (2006) 86(8):853-64. doi: 10.1038/labinvest.3700443.

3. Gopallawa I, Kuek LE, Adappa ND, Palmer JN, Lee RJ. Small-Molecule Akt-Activation in Airway Cells Induces No Production and Reduces Il-8 Transcription through Nrf-2. *Respir Res* (2021) 22(1):267. Epub 2021/10/21. doi: 10.1186/s12931-021-01865-y.

4. Valley HC, Bukis KM, Bell A, Cheng Y, Wong E, Jordan NJ, et al. Isogenic Cell Models of Cystic Fibrosis-Causing Variants in Natively Expressing Pulmonary Epithelial Cells. *J Cyst Fibros* (2019) 18(4):476-83. Epub 2018/12/20. doi: 10.1016/j.jcf.2018.12.001.

5. McMahon DB, Workman AD, Kohanski MA, Carey RM, Freund JR, Hariri BM, et al. Protease-Activated Receptor 2 Activates Airway Apical Membrane Chloride Permeability and Increases Ciliary Beating. *FASEB J* (2018) 32(1):155-67. doi: 10.1096/fj.201700114RRR.

6. Grynkiewicz G, Poenie M, Tsien RY. A New Generation of Ca2+ Indicators with Greatly Improved Fluorescence Properties. *J Biol Chem* (1985) 260(6):3440-50.

7. Lund VJ, Mackay IS. Staging in Rhinosinusitus. *Rhinology* (1993) 31(4):183-4. Epub 1993/12/01.

8. Lund VJ, Kennedy DW. Staging for Rhinosinusitis. *Otolaryngol Head Neck Surg* (1997) 117(3 Pt 2):S35-40. Epub 1997/10/23. doi: 10.1016/S0194-59989770005-6.

9. Quintanilla-Dieck L, Litvack JR, Mace JC, Smith TL. Comparison of Disease-Specific Quality-of-Life Instruments in the Assessment of Chronic Rhinosinusitis. *Int Forum Allergy Rhinol* (2012) 2(6):437-43. Epub 2012/06/15. doi: 10.1002/alr.21057.
